# National and Hospital Level Antimicrobial Consumption Patterns in Kenya

**DOI:** 10.3390/antibiotics15060587

**Published:** 2026-06-09

**Authors:** Kizito M. Mariita, Loice A. Ombajo, Christine M. Ngacha, Bramuel Tongola, Vallarie Khamira, Rosemary Njogu, Karim Wanga, Lydia Momanyi, Joram Andrew, Edwin Otieno, Marion N. Ong’ayo, Salome Karuri, Lucy Ochola, Neto Obala, Margaret O. Oluka, Emmanuel Tanui, Silas C. Kandie, Sarah Kibira, Dorothy Aywak, Swabra Omar

**Affiliations:** 1 Department of Pharmacy, The Nairobi Hospital, Nairobi P.O. Box 30026-00100, Kenya; kizitomochama@nbihosp.org; 2 Department of Clinical Medicine and Therapeutics, University of Nairobi, Nairobi P.O. Box 19676-00202, Kenya; 3 Center for Epidemiological Modelling and Analysis, University of Nairobi, Nairobi P.O. Box 19676-00202, Kenya; christinem@uonbi.ac.ke (C.M.N.); joram.andrew@cema.africa (J.A.); edwin@uonbi.ac.ke (E.O.); 4 Pharmacy and Poisons Board, Nairobi P.O. Box 27663-00506, Kenya; bramuel@ppb.go.ke (B.T.); vallarie@ppb.go.ke (V.K.); rosemary@ppb.go.ke (R.N.); 5 Inter-Governmental Authority on Development, Nairobi P.O. Box 551-50100, Kenya; karim.wanga@igad.int; 6 Nakuru County Teaching and Referral Hospital, Nakuru P.O. Box 71-20100, Kenya; momanyilydia22@gmail.com; 7 Mbagathi County Referral Hospital, Nairobi P.O. Box 20725-00202, Kenya; ongayomarion@gmail.com; 8 Thika Level V Hospital, Thika P.O. Box 227-01000, Kenya; sal.karuri@gmail.com; 9 Machakos County Referral Hospital, Machakos P.O. Box 19-90100, Kenya; lucyochola@gmail.com; 10 Jaramogi Oginga Odinga Teaching and Referral Hospital, Kisumu P.O. Box 849-40100, Kenya; drnetoobala@jootrh.go.ke; 11 Department of Pharmacology, Clinical Pharmacy and Pharmacy Practice, University of Nairobi, Nairobi P.O. Box 19676-00202, Kenya; moluka@uonbi.ac.ke; 12 Kenya National Public Health Institute, Nairobi P.O. Box 31-00202, Kenya; tanuikip@yahoo.com; 13 Kisii Teaching and Referral Hospital, Kisii P.O. Box 92-40200, Kenya; silas.cherogony@ktrh.co.ke; 14 Nyeri County Referral Hospital, Nyeri P.O. Box 27-10100, Kenya; kibirasarah@gmail.com; 15 Kenyatta National Hospital, Nairobi P.O. Box 20723-00202, Kenya; daywak@knh.or.ke; 16 Coast General Teaching and Referral Hospital, Mombasa P.O. Box 90231-80100, Kenya; swabraomar3@gmail.com

**Keywords:** antimicrobial consumption, AWaRe, drug utilization, Kenya, antimicrobial stewardship

## Abstract

**Background**: Robust antimicrobial consumption monitoring and correlation with antibiotic resistance trends is critical to informing evidence-based antimicrobial stewardship and is recommended by the World Health Organization Global Action Plan on the containment of antimicrobial resistance. We estimated national and hospital-level antibacterial consumption patterns in Kenya. **Materials and methods**: National consumption data (January 2023–December 2024) was derived from aggregated import and donation permits at the Pharmacy and Poisons Board and standardized using the WHO ATC/DDD Index 2025. Consumption data for 2020 and 2021 that had been previously submitted by Kenya to WHO GLASS was retrieved and incorporated to allow a description of trends. Hospital-level data was collected from ten facilities across the country for the period from January 2024 to April 2025. Quality of use was evaluated using the WHO Access, Watch, and Reserve (AWaRe) categorisation with high-consumption antibiotics identified using the Drug Utilization 75% metric. **Results**: There was a gradual increase in national antibiotic consumption from 14.3 DID in 2020 to 22.2 DID in 2024. Oral formulations accounted for more than three quarters of antibiotics consumed. Access category antibiotic consumption ranged from 50.4% to 56.9% nationally and was 61.2% at hospital level. The national consumption of Watch antibiotics increased from 38.9% in 2020 to 46.1% in 2023 and declined to 40.7% in 2024. Amoxicillin and amoxicillin/clavulanic acid were the most commonly consumed antibiotics nationally in 2020 (40%), 2021 (27%) and 2024 (33%). Azithromycin was the most commonly consumed antibiotic in 2023 (27%), rising from 10% in 2020. Among parenteral antibiotics, benzylpenicillin was the most commonly consumed in 2020 and 2021, while ceftriaxone was the most commonly consumed agent in 2023 (24%) and 2024 (41%). At hospital level, ceftriaxone accounted for 56.5% of parenteral antibiotic use in county referral hospitals. **Conclusions**: Kenya’s antibacterial consumption is increasing. Use of Access antibiotics remains below the WHO target of 60%. The increasing use of Watch antibiotics, and in particular ceftriaxone and azithromycin, needs to be addressed to support Kenya’s efforts against antimicrobial resistance.

## 1. Introduction

The effectiveness of antimicrobials is increasingly threatened by rising levels of antimicrobial resistance (AMR), posing a major risk to global health, food security, and economic development. Inappropriate and excessive antibiotic use is the primary driver of AMR, and surveillance of antibiotic use is a central pillar of the World Health Organization (WHO) Global Action Plan (GAP) on AMR [1].

Monitoring antimicrobial consumption (AMC) provides critical insights into patterns of use over time and across different settings. Typically reported periodically (annually, biannually, quarterly, or before and after interventions), AMC data are used to assess trends, compare consumption between departments, institutions, countries, and regions, and evaluate the impact of antimicrobial stewardship interventions [2]. The most widely used method of measuring AMC is the WHO Anatomical Therapeutic Chemical/Defined Daily Dose (ATC/DDD) system, which enables comparisons across settings by expressing consumption as DDD per 1000 inhabitants per day (DID) at the population level or DDD per 100 occupied bed days (OBD) at facility level. Alternative approaches include prescription-based metrics such as the number of prescriptions and days of therapy [1].

Global and regional targets for AMC have been established to guide antimicrobial stewardship efforts. The WHO AWaRe classification initially set a target of at least 60% of antibiotic consumption from the Access category [3,4]. This was increased to 70% by 2030 in the 2024 United Nations General Assembly political declaration on antimicrobial resistance, reflecting a stronger emphasis on narrower-spectrum antibiotic use [5]. While many developed countries have established robust AMC surveillance systems with clearly defined national targets [6], most low- and middle-income countries are still in the process of developing and strengthening such systems [7]. The European Center for Disease Prevention and Control 2019 (ECDC) AMC findings informed two key targets: reducing overall population-weighted antibiotic consumption by 20% (from 19.9 DID to 15.9 DID by 2030), and ensuring that at least 65% of antibiotics consumed are from the Access group [8]. In 2023, ECDC AMC was 20.0 DID, representing a 1% increase from the 2019 baseline and remaining above their 2030 target. In the same year, the mean proportion of WHO Access antibiotics consumed in the EU was 61.5%, falling below both the EU and the revised global 2030 targets [8]. These observations highlight persistent gaps in optimizing antimicrobial use globally.

In Kenya, national AMC was first estimated for the period 2016–2018 using data from the Kenya Medical Supplies Agency and selected wholesalers and distributors. Pharmacy-level data was obtained from 10 community and 15 hospital pharmacies that also had antimicrobial susceptibility-testing laboratories. The average national AMC during this period was 8.8 DID [9]. The five most commonly consumed antimicrobials were amoxicillin, sulfamethoxazole/trimethoprim, ampicillin/cloxacillin, erythromycin, and doxycycline, accounting for more than 58% of the total consumption.

A more comprehensive national AMC assessment was subsequently conducted by the Pharmacy and Poisons Board (PPB) for the period 2018–2021. Amoxicillin was the most commonly consumed oral antibiotic in 2018 (71.08%), 2019 (50.54%), and 2020 (32.89%). Among parenteral antibiotics, benzylpenicillin ranked highest in 2018, 2019, and 2021, while ceftriaxone topped in 2020 [10]. These findings underscored changing consumption patterns and the growing use of broad-spectrum agents.

Given the limited number of published AMC studies from countries in sub-Saharan Africa, continued surveillance in Kenya is essential for understanding the evolving consumption patterns and correlating these trends with AMR data to guide policy and practice.

The objective of this surveillance was to determine the national and hospital-level consumption of antibacterials in Kenya and to describe the patterns of antibiotic consumption in order to inform national antimicrobial stewardship interventions.

## 2. Methodology

### 2.1. Study Design and Setting

The study estimated AMC at national and hospital levels using the WHO GLASS-Antimicrobial Consumption (GLASS-AMC) methodology [11].

For national level consumption, data was derived from aggregated sources; specifically import and donation permit records obtained from the national Pharmacy and Poisons Board (PPB). PPB is Kenya’s statutory regulatory body responsible for controlling medicine importation and registration. Import data accounts for approximately 95% of medical products in the country and served as a robust proxy for nationwide consumption during the surveillance period, recognizing that national consumption data does not fully reflect actual patient-level use. Data on antimicrobials for systemic use, encompassing J01 (antibacterials for systemic use), A07AA (antibiotics (intestinal anti-infectives)), P01AB (nitroimidazole derivatives); J02 (antifungals), J05 (antivirals), P01B (antimalarials), and J04 (antimycobacterials) for the period from January 2023 to December 2024 was retrieved from electronic records. To understand trends, we compared the current period of surveillance with consumption data for 2020 and 2021 that had been previously submitted by Kenya to WHO GLASS. Dasta from 2022 was not captured as surveillance activities under the current funding cycle began retrospectively from the most recent years. Resource and regulatory timelines limited completion of data abstraction for 2022 during the study period.

For hospital-level AMC, data was collected from ten purposively selected hospitals supported under the Fleming Fund Kenya country grant. These included one national referral, eight county referrals and one private tertiary hospital. The facilities are high-volume referral facilities serving large catchment populations and therefore provide an important overview of antimicrobial consumption patterns across major public and private healthcare settings in Kenya.

### 2.2. Data Sources

Data was obtained from commercial and donation import permits accessed through the Kenya Trade Network Agency electronic Single Window System, an online platform used for submission, processing, and archiving of trade-related documentation. The retrieved permits were subjected to rigorous data abstraction and entry into the Kenya Surveillance System for Antimicrobial Consumption (KESAC, Pharmacy and Poisons Board, Nairobi, Kenya), a national web-based tool [12] designed in line with WHO AMC data collection requirements [11]. Permits for medical devices, investigational products, raw materials, and topical antimicrobials were excluded. Population denominators were derived from United Nations World Population Prospects, which provide standardized and internationally comparable estimates [13].

Hospital-level data was retrieved from manual and electronic records at the pharmacies and bulk medication stores, which track the distribution of systemic antibiotics across the various hospital departments. This data reflected antimicrobials dispensed to both outpatients and to patients admitted in the hospital. Records of hospital occupancy were derived from the health facilities’ medical statistics. Each hospital data was collected during the period from January 2024 to April 2025.

### 2.3. Data Abstraction

Data abstraction from the import permits and entry into the KESAC tool focused on product-level variables necessary for the accurate calculation of Defined Daily Doses (DDD). These variables included the active pharmaceutical ingredient, product name, strength, pack size, route of administration, and the total number of packages imported. Missing information on active ingredients or formulations was supplemented using the PPB electronic common technical document database (a public repository of electronically registered medicine product details, including active ingredient and formulation information) [14], and the national drug index.

Unit quantities consumed at hospital level were entered in an excel spreadsheet (Microsoft corporation, Redmond, WA, USA; Microsoft excel 2016). The spreadsheet listed all antimicrobials in the hospitals, including available formulations and strengths. Drug volumes consumed for each antimicrobial were calculated at the hospital level and entered into the spreadsheet alongside the hospital occupied-bed days (OBD). The assigned WHO DDD values for each drug and formulation were incorporated with automated calculation of antimicrobial consumption which was expressed as total DDDs and DDD/100 OBD.

The WHO ATC/DDD Index 2025 version [15] was used to classify the antimicrobials. Each antimicrobial substance was assigned its corresponding ATC 5th level code, which identifies the specific chemical substance [16] and then categorized according to the WHO AWaRe framework of Access, Watch and Reserve [3].

### 2.4. Antimicrobial Consumption Measurement Metrics

#### 2.4.1. Total Consumption

Consumption estimates were standardized using the DDD. The total number of DDDs for each product was calculated by multiplying the quantity of active substance by the WHO DDD value.

National level consumption was then expressed as the DDD per 1000 Inhabitants per Day (DID) metric, with the population based on the United Nations estimates [13]:

DID = (Total DDD × 1000)/(Population × 365)

#### 2.4.2. AWaRe Categorization

The proportions of antibiotics consumed within various AWaRe categories were calculated relative to the total systemic antibacterial consumption (J01, A07AA, P01AB) and compared to the global target set by the WHO.

#### 2.4.3. Drug Utilization 75% (DU75%)

The DU75% metric was used to identify the antibiotics accounting for the highest consumption at both national and hospital levels. This metric identifies the antibiotics contributing most substantially to overall consumption. Antibiotic consumption values were ranked in descending order. Antibiotics cumulatively accounting for 75% of total consumption constituted the DU75 segment.

## 3. Results

### 3.1. Trend of Total AMC

The national consumption of antibacterials (J01) was 14.3 DID, 19.8 DID, 20.7 DID, 22.20 DID in 2020, 2021, 2023, and 2024 respectively. Oral antibiotics comprised over 90% of the antibiotics consumed in 2023 and 2024. Consumption of antimalarials (P01B) increased from 1.3 DID to 2.7 DID while that of antivirals for systemic use (J05) decreased from 7.8 DID to 7.1 DID between 2023 and 2024 (Table 1).

### 3.2. Consumption by WHO AWaRe Classification

National level consumption was the highest in the Access category, accounting for 50.6% (2020), 56.9% (2021), 52.1% (2023), and 50.4% (2024). Watch category consumption increased from 38.90% (2020) to 46.1% (2023). Reserve category consumption remained low while that in the Not Recommended category reduced from a high of 10.4% in 2020 to 1.4% in 2023 (Table 2).

**Figure 1:**
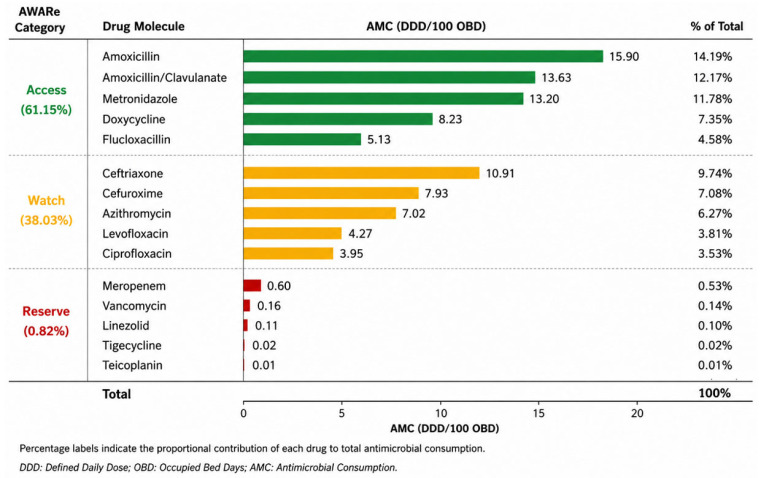
Mean antibacterial consumption across participating hospitals by WHO AWaRe category expressed as DDD/100 occupied bed days (OBD) and proportional contribution to total antibacterial consumption.

The mean antibacterial consumption across the participating hospitals was 112.0 DDD/100 occupied bed days (OBD) with Access and Watch category antibacterials accounting for 61.15% and 38.03% consumption, respectively. Amoxicillin (14.2%), amoxicillin/clavulanate (12.2%), and metronidazole (11.8%) were the most commonly consumed Access antibiotics while ceftriaxone (9.7%) was the most common in the Watch category. Among the Reserve antibiotics, meropenem was the most commonly consumed (Figure 1).

### 3.3. National Level Drug Utilization 75%

**Figure 2:**
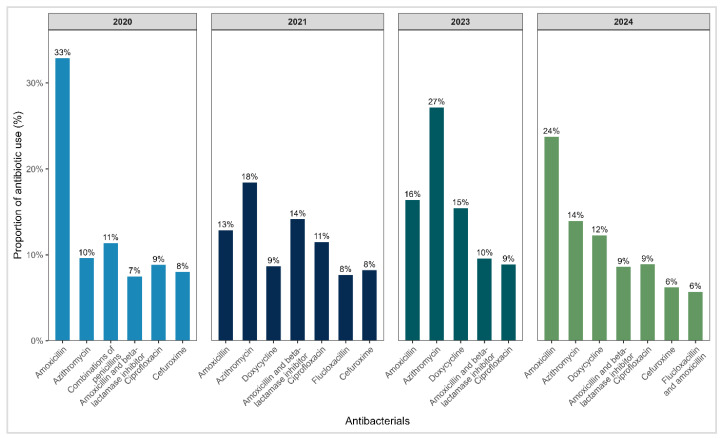
National DU75% consumption patterns of oral antibiotics across the surveillance years.

Nationally, amoxicillin had the highest consumption in 2020 (33%) and 2024 (24%), while azithromycin had the highest consumption in 2021 (18%) and 2023 (27%). Doxycycline was the third most commonly consumed antibiotic in 2023 (15%) and 2024 (12%). Consumption of amoxicillin–beta-lactamase inhibitor combinations ranged from 7% (2020) to 14% (2021), and decreased to 10% (2023) and 9% (2024) (Figure 2).

**Figure 3:**
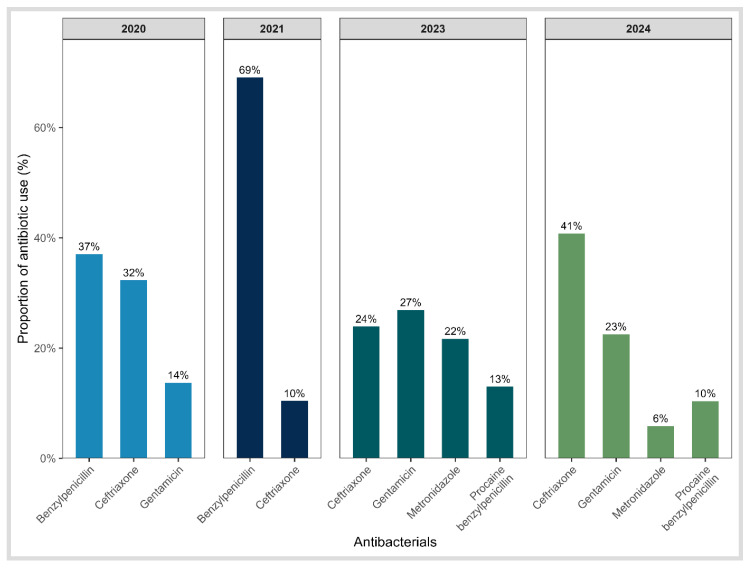
National DU75% consumption patterns of parenteral antibiotics across the surveillance period.

Benzylpenicillin was the most commonly consumed parenteral antibiotic nationally in 2020 and 2021, accounting for 37% and 69% respectively. During the same period, ceftriaxone had the second-highest consumption at 32% and 10%, respectively. However, in 2023 and 2024, gentamicin and ceftriaxone were the most commonly consumed parenteral antibiotics at 27%and 41%, respectively. Gentamicin had the second-highest consumption in 2024 (23%) (Figure 3).

### 3.4. Drug Utilization 75% in Different Hospitals

Amoxicillin/clavulanic acid and cefuroxime were the most commonly consumed oral antibiotics in public national referral and private hospitals while the public country referral hospitals consumed more of amoxicillin (25.2%), oral metronidazole (15.3%) and doxycycline (11.9%). Consumption of oral azithromycin was relatively similar across the hospital categories. Among the parenteral antibiotics, ceftriaxone consumption in the public hospitals was high (56.5%). Consumption in the private hospital was distributed across a wider range of parenteral antibiotics. Consumption of meropenem in the national referral hospital and the private hospital was 7.1% and 9%, respectively (Table 3).

### 3.5. Consumption by WHO ATC Classification

**Figure 4:**
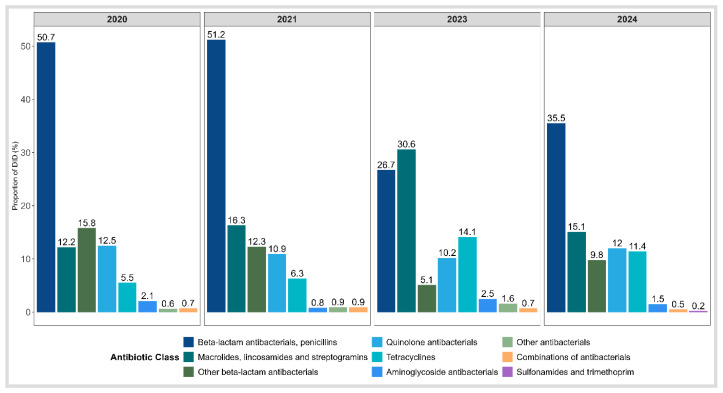
National antibacterial consumption by WHO ATC classification across the surveillance years.

At the national level, beta-lactam antibacterials, penicillins J01C, were the most commonly consumed class in 2020 (51%), 2021 (51%), and 2024 (36%). Macrolides, lincosamides, and streptogramins had the highest consumption in 2023 and the second highest in 2021 and 2024. Other beta-lactam antibacterials J01D ranked second in 2020 and third in 2021. Quinolone consumption was stable at 10% to 12% over the four years. Aminoglycoside use was generally low (Figure 4).

**Figure 5:**
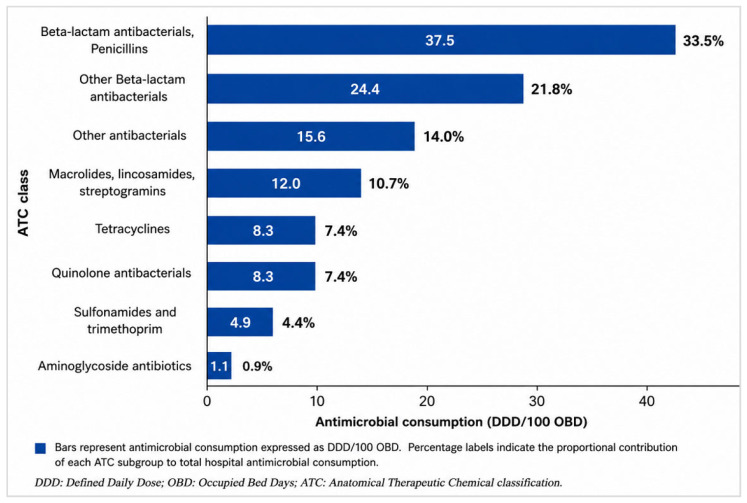
Mean antibacterial consumption across participating hospitals by ATC classification expressed as DDD/100 OBD (the proportional contribution to total antibacterial consumption is indicated on the right of each bar).

At hospital level, beta-lactam antibacterials, penicillins J01C (33.5%), and other beta-lactam antibacterials J01D (21.7%) had the highest consumption. Aminoglycosides, sulphonamides and trimethoprim, quinolones and tetracyclines were the least consumed (Figure 5).

## 4. Discussion

Over the review period, Kenya’s national AMC increased by 55%, rising from 14.3 DID in 2020 to 22.2 in 2024. This consumption is above the global median of 18.3 DID reported across the countries that participate in global surveillance [17]. Kenya’s consumption pattern mirrors that of LMICs in Asia, where increases have been observed following the COVID-19 pandemic [17]. Compared with other countries in sub-Saharan Africa, Kenya’s 2024 AMC is lower than Uganda’s 29.02 DID in 2021 [18] and Tanzania’s 38.27 ± 5.17 DID [19], and substantially higher than Ethiopia’s 11.34 DID [20] in 2022. The increase in consumption over the years is concerning. This trend highlights the need for stricter enforcement of dispensing regulations in private pharmacies, alongside public awareness campaigns, to reduce inappropriate demand and enhance community-level antimicrobial stewardship.

The 51% national consumption of antibiotics in the Access category falls below the ≥60% target recommended by the WHO for national antibiotic consumption [3,4] and the ≥70% target set for 2030 by the United Nations General Assembly [5] as part of global commitments to optimize antibiotic use and curb antimicrobial resistance. The national consumption is close to the global proportion in 2022 where 52.7% of the antibiotics consumed were from the Access group [17]. Of note, only 58% and 31.7% of countries that reported AMC to GLASS in 2022 met the 2023 and 2030 targets, respectively [17]. Rwanda, Ivory Coast, South Africa, Botswana and Ethiopia were the African countries that met the UNGA 2030 70% Access target [17]. For Rwanda and South Africa, this finding may reflect the fact that the data only represented the public sector, which primarily serves a population segment with limited access to higher classes of antibiotics. In addition to import data, locally manufactured antibiotics accounted for more than 25% of Ethiopia’s antibiotic consumption. The majority of these are likely oral formulations within the Access category, owing to their ease of manufacture and less stringent regulatory requirements [20].

A few surveys indicate that Kenya’s consumption of Access antibiotics may be lower than that of its neighbours Tanzania and Uganda. In Uganda, Access antibiotics accounted for 72% of consumption in 2028 and 65% in 2021 and exceeded 60% in Tanzania during the period 2020–2022 [18,19]. While Kenya and Uganda relied on import data, Tanzania included data from local manufacturers and from its central medical stores. Incorporating central medical store data can tilt consumption patterns toward government-procured medicines; these are typically restricted to national essential medicines lists, which generally over-represent Access antibiotics.

The high proportion of Watch antibiotics is partly driven by increased consumption of azithromycin, which was the most commonly consumed antibiotic nationally in 2021 and 2023. This may reflect prescribing practices during and immediately following the COVID-19 pandemic, when azithromycin was widely used empirically for respiratory tract infections despite limited evidence of benefit against viral illness [21]. Additionally, community-level access to antibiotics without prescription and increased public demand for antibiotics may have exacerbated these trends [7]. Similar increases in azithromycin use during the COVID-19 pandemic period have been observed globally [17].

Patterns in parenteral antibiotic use showed a shift from benzylpenicillin to ceftriaxone over time. While benzylpenicillin was the most commonly consumed injectable antibiotic in 2020 and 2021, ceftriaxone was the leading parenteral antibiotic in 2023 and 2024. This shift suggests an increasing reliance on broader-spectrum antibiotic therapy in empiric treatment and widespread availability of ceftriaxone in healthcare facilities. Similar prescribing patterns have been reported in other Kenyan studies, where ceftriaxone and azithromycin are among the most commonly used antibiotics in hospital and community settings [21,22]. The high consumption of ceftriaxone is concerning for two reasons. First, widespread use of broad-spectrum antibiotics is associated with higher odds of selection of resistant pathogens [23] and this may explain the rise in third-generation cephalosporin-resistant Enterobacterales [24]. Secondly, given the high resistance rates, ceftriaxone may no longer be an appropriate empiric choice for multiple common infections in this setting. This underscores the need to strengthen antimicrobial stewardship interventions and promote use of Access antibiotics where clinically appropriate.

The consumption of Reserve category antibiotics nationally was low, accounting for only 0.05% to 0.1% of total consumption. At the hospital level, the proportion was slightly higher at 0.8%, which is expected given that most of these antibiotics are parenteral and primarily administered in the inpatient setting. In 2023, the European Union population-weighted mean proportion of Reserve antibiotics in hospitals was 5.40%, ranging from 0.65% in Finland to 15.90% in Greece [8]. Our findings suggest limited reliance on last-resort agents, potentially helping to preserve their effectiveness by slowing the development of resistance. However, the low utilization may also reflect lack of access to Reserve antibiotics in resource-limited settings. Such access is critical if AMR-related mortality is to be reduced. The 2022 WHO report on AMC also highlights that eight of the 60 countries (13.3%) that reported no use of antibiotics in the Reserve category were from low-income and LMICs. For the countries that reported any use of Reserve antibiotics, this was low, accounting for 0.2% of total antibiotic use [17].

Despite the consumption of Not classified or Not recommended antibiotics being relatively low in 2021 and 2023, their consumption exceeded 5% in 2020 and 2024. It is imperative that the use of the Not recommended antibiotics is investigated as their efficacy may not be proven and they are also not recommended by WHO or global guidelines. Many of these are often unjustified fixed-dose combinations of broad-spectrum drugs with a potential to promote AMR and should be excluded from the list of registered products.

Beta-lactam antibacterials, penicillins J01C, had the highest consumption at national and hospital level. Macrolides consumption was conspicuously high at national level and even surpassed that of penicillins in 2023, largely driven by the widespread increases in azithromycin use globally during and immediately after the COVID-19 pandemic. This is consistent with the 2022 WHO global report where the median proportional use of extended-spectrum penicillins and combinations of penicillins, including beta-lactamase inhibitors, was the highest at 31.3% followed by macrolides at 14.7% [17]. A similar pattern is observed across the East African region where the consumption of penicillins was consistently high in Uganda, Tanzania, Ethiopia, and Zambia. The consumption of quinolones, cephalosporins and macrolides was variable in these countries, possibly reflecting differences in prescribing practices or disease patterns [18,19,20,25].

The consumption of other beta-lactam antibacterials (J01D) and other antibacterials (J01X) was higher at hospital than at national level. A similar pattern was seen in the European hospital sector data where the most commonly consumed subgroup in 2023 was penicillins (34%), followed by cephalosporins and other beta-lactams (28%), and other antibacterials 12% [8]. Consumption of ceftriaxone, cefuroxime and metronidazole was common, and it is important to analyze the indications for which these antibiotics are used. For instance, the fact that cefazolin consumption was almost negligible, may be an indicator that other antibiotics are being used for surgical prophylaxis. Similarly, the oral Access antibiotic nitrofurantoin, which has excellent activity against Escherichia coli and is recommended for lower urinary tract infections, did not feature in the DU75% at either level, despite reports of high prevalence of UTI morbidity in the country [26,27]. Both cefazolin and nitrofurantoin are relatively affordable antibiotics and efforts should be made to make them available. Dissemination and implementation of Kenya’s treatment guidelines [28], which recommend the use of such simple yet effective molecules, is an essential step in reversing this trend.

Kenya has made important progress in implementing its National Action Plan on Antimicrobial Resistance [2] with set up of one health antimicrobial stewardship structures at national and sub-national levels, development of national antibiotic guidelines [27], and strengthening of surveillance systems. However, the continued increase in Watch antibiotic consumption suggests that further stewardship strengthening, improved diagnostic capacity, and enhanced regulation of antimicrobial dispensing remain necessary. Although Kenyan regulations require antibiotics to be dispensed with a prescription, over-the-counter access to antibiotics in community pharmacies and informal medicine outlets remains a recognized challenge in many low- and middle-income settings [7,28], contributing to inappropriate community-level antibiotic use.

## 5. Conclusions

Kenya’s antibacterial consumption is increasing and use of Access category antibiotics is below the targets set by the WHO. The increasing use of Watch antibiotics, particularly ceftriaxone and azithromycin, underscores the need to strengthen antimicrobial stewardship and improve awareness and adherence to treatment guidelines. Antibiotic dispensing regulations should also be enforced alongside enhanced antimicrobial consumption and resistance surveillance in Kenya.

## 6. Limitations

This study did not include consumption of locally manufactured antimicrobials; however, these are likely to contribute to a very small fraction of the country’s total antimicrobial consumption. We used aggregated data sources, which serve as proxy measures of antimicrobial use but may overestimate or underestimate true antimicrobial use due to factors such as wastage, delayed utilization, or variations in supply chain practices. The DDD methodology used may not accurately estimate antimicrobial use among pediatric populations. However, it can allow for comparison of trends, where the mix of the adult and pediatric population is stable over time. Pediatric antibiotic consumption was included because medicines for both adult and pediatric patients were dispensed through shared pharmacy systems. As such, pediatric consumption could not be reliably separated from adult consumption data. In addition, hospital-level data were obtained from a limited number of facilities and covered a relatively short period of 16 months.

## Figures and Tables

**Table 1:** National antimicrobial consumption trends in Kenya expressed as Defined Daily Doses per 1000 inhabitants per day (DID).

	Consumption DID			
Category	2020 *	2021 *	2023	2024
Antibacterials (J01)	14.3 (100%)	19.8 (100%)	20.7 (58.3%)	22.2 (53.1%)
Oral	12.2 (85.2%)	14.2 (71.4%)	18.8 (90.8%)	20.7 (92.9%)
Parenteral	2.1 (14.8%)	5.7 (28.6%)	1.9 (9.2%)	1.6 (7.1%)
Antibacterials (A07AA, P01AB)	-	-	2.0 (5.6%)	1.3 (3.1%)
Antimalarials (P01B)	-	-	1.3 (3.5%)	2.7 (6.5%)
Antimycotics and antifungals for systemic use (J02, D01B)	-	-	1.1 (3.1%)	2.0 (4.8%)
Antivirals for systemic use (J05)	-	-	7.8 (22.1%)	7.1 (17%)
Drugs for the treatment of tuberculosis (J04A)	-	-	1.7 (4.8%)	5.8 (13.9%)
Non-Antimicrobial (J04B, P01A, Without ATCs)	-	-	0.9 (2.6%)	0.7 (1.6%)

* 2020 and 2021 data was available for J01 only.

**Table 2:** National antibacterial consumption by WHO AWaRe categorisation for the surveillance period.

WHO AWaRe Classification				
Category	2020	2021	2023	2024
Access	7.23 (50.60%)	11.3 (56.92%)	11.8 (52.08%)	11.84 (50.42%)
Watch	5.56 (38.9%)	7.81 (39.36%)	10.43 (46.05%)	9.56 (40.69%)
Reserve	0.01 (0.06%)	0.01 (0.05%)	0.01 (0.03%)	0.01 (0.06%)
Not recommended	1.49 (10.44%)	0.73 (3.66%)	0.32 (1.4%)	1.92 (8.16%)
Uncategorized	-	-	0.1 (0.44%)	0.16 (0.67%)

**Table 3:** DU75% oral and parenteral antibacterial consumption patterns across county referral, national referral and private hospitals.

	Public County Referral Hospitals	Public National Referral Hospital	Private Hospital
Drug Utilization 75% Oral antibiotics	Amoxicillin 25.2% Metronidazole 15.3% Doxycycline 11.9% Amoxicillin/clavulanic Acid 9.0% Azithromycin 8.8% Cotrimoxazole 7.2%	Amoxicillin/clavulanic Acid 39.8% Cefuroxime 10.2% Clindamycin 9.0% Metronidazole 9.0% Azithromycin 8.9%	Amoxicillin/clavulanic Acid 27.5% Cefuroxime 23.8% Levofloxacin 7.2% Azithromycin 7.1% Amoxicillin 4.6% Doxycycline 4.5% Clarithromycin 4.2%
Drug Utilization 75% Parenteral antibiotics	Ceftriaxone 56.5% Metronidazole 17.9% Flucloxacillin 5.1%	Ceftriaxone 36.1% Amoxicillin/clavulanate 16.1% Metronidazole 15.0% Meropenem 7.1%	Ceftriaxone 20.9% Cefuroxime 16.0% Amoxicillin/clavulanate 11.8% Meropenem 9.0% Levofloxacin 6.8% Metronidazole 5.4% Piperacillin/tazobactam 4.2% Cefepime 3.0%

## Data Availability

Replication data for this study is available with publication at the Harvard Dataverse by request to the Principal Investigator (L.A.O.) based on an approved proposal, at https://doi.org/10.7910/DVN/WNLMAW.
